# Cephalexin-associated Achilles Tendonitis: Case Report and Review of Drug-induced Tendinopathy

**DOI:** 10.7759/cureus.3783

**Published:** 2018-12-27

**Authors:** Philip R Cohen

**Affiliations:** 1 Dermatology, San Diego Family Dermatology, San Diego, USA

**Keywords:** achilles, cephalexin, drug, fluoroquinolone, medication, rupture, tendon, tendonitis, tendinopathy, toxic

## Abstract

Tendon disorders include tenosynovitis or tendonitis, tendinosis, and tendon rupture. Tendinopathy associated with drug administration has been associated with the systemic or local administration of several medications. A 90-year-old man who developed toxic tendinopathy after receiving cephalexin 500 mg twice daily has been described. Unilateral pain of his left Achilles tendon pain during walking appeared three weeks after starting the antibiotic. The drug was stopped after four weeks of treatment; within one week after discontinuing the cephalexin, all tendonitis symptoms spontaneously resolved. Drug-induced tendinopathy has most commonly been associated with fluoroquinolones, statins, glucocorticoids, and aromatase inhibitors. In addition, other systemic agents have caused tendinopathy; they include amlodipine, anabolic steroids, antiretrovirals, isotretinoin, renin-angiotensin II receptor antagonists, rituximab, and sitagliptin. Albeit less frequent, other oral antibiotics, including cephalosporins, azithromycin, and sulfonamides, have also been associated with toxic tendinopathy. Also, injections of collagenase *Clostridium histolyticum*, corticosteroids, and polidocanol have been followed by tendon rupture. The features of tendinopathy associated with drug treatment are summarized and their postulated mechanisms of pathogenesis are reviewed. The onset of tendon pain following the initiation of treatment with a new medication, especially if the agent has previously been associated with drug-induced tendonitis, tendinosis, or tendon rupture, should prompt the consideration of drug-associated toxic tendinopathy.

## Introduction

Tendon disorders can range from tendonitis to rupture [1-3]. Several medications have been implicated in toxic tendinopathy associated with drug administration [1-20]. A man with cephalexin-associated tendonitis is described and agents that have been implicated in drug-induced tendinopathy are reviewed.

## Case presentation

A 90-year-old man presented for evaluation of asymptomatic pigmented lesions on his soles. His past medical history is significant for prostate cancer. He receives leuprolide acetate (Lupron) depot suspension 22.5 mg injection every three months.

Cutaneous examination showed black macules on his feet. An 8 x 8-mm black macule was present on his left plantar foot near the heel. A 10 x 10-mm black macule was present on the right plantar midfoot.

A 3-mm punch biopsy was performed at each site. Antibiotic prophylaxis, cephalexin 500 mg twice daily, was prescribed for 15 days. Topical mupirocin ointment (2%) was applied to the biopsy sites three times daily.

He returned for suture removal after two weeks. The left foot showed a combined (blue and junctional) nevus that was present in the lateral margins of the specimen; since this is a benign lesion, no further treatment was necessary. However, the right foot showed a junctional nevus with dysplastic features that also extended to the specimen’s lateral margins; the dermatopathologist recommended an additional biopsy.

A broader biopsy, using the shave technique, was done and included most of the residual pigmented lesions on his right foot. Cephalexin 500 mg twice daily was continued for an additional two weeks. He also continued to apply the mupirocin ointment (2%) to the area three times daily.

After an additional week of cephalexin—his third consecutive week receiving the antibiotic—he began to experience tenderness of his left Achilles tendon when walking. He was scheduled to return to the office one week later. He continued to take the antibiotic and his tendon pain progressively increased.

Follow-up examination, two weeks after the second biopsy (and four weeks after starting cephalexin), showed partial healing of the biopsy site on his left foot; there was neither inflammation nor tenderness. Pathology evaluation of the larger biopsy specimen demonstrated a benign compound nevus, having features consistent with congenital onset, and without any atypia. Based on the revised diagnosis, no further intervention was necessary for the residual lesion that extended to the deep and lateral margins of the specimen.

Examination of his Achilles tendons was also performed. His right tendon was asymptomatic. Similarly, at rest, his left tendon had no pain. However, on ambulation, the left Achilles tendon was very tender.

Cephalexin was discontinued; he had received four weeks of treatment. Ibuprofen was prescribed for symptomatic relief; however, the patient decided not to initiate oral therapy with the nonsteroidal anti-inflammatory drug. Within one week after stopping cephalexin, the left Achilles tendon pain spontaneously resolved and has not recurred. Subsequently, during the evaluation of his Achilles tendons two weeks after cephalexin had been withdrawn, there was no pain when walking.

## Discussion

Tendinopathy includes tenosynovitis or tendonitis, tendinosis, and tendon rupture. Tenosynovitis describes tendinopathy with inflammation of the paratendinous tissues. However, the term tendonitis also designates—in both the medical literature and nonmedical publications—the clinical presentation of pain affecting a tendon and the associated changes observed on magnetic resonance images [1-3].

Tendinosis refers to the macroscopic and microscopic changes associated with chronic tendon degeneration. Gross examination shows tan or brown discoloration of a rough and irregularly thickened tendon. Microscopic changes include alteration of the extracellular matrix composition, distortion—with or without apoptosis—of the tenoblasts and tenocytes, fibril disorganization, and neovascularization [1-3].

Tendon rupture may be partial or complete. Extrinsic muscle contractions (that subject the tendon to oblique tensile forces) during rapid repeat movements provide the greatest risk of tendon rupture. In addition, tendons that are deteriorated are at an increased risk to rupture [1-3].

Drug-induced tendinopathy, also referred to as toxic tendinopathy, has been associated with the systemic or local administration of several medications. Fluoroquinolones are the most notable culprits. However, other systemic agents that have more commonly resulted in either tendonitis or tendon rupture include statins, glucocorticoids, and aromatase inhibitors [1-3].

Fluoroquinolone tendon toxicity (tendonitis and tendon rupture) can be observed with all members of this class of antibiotics, regardless of the administration route or the dosage. The incidence of fluoroquinolone tendinopathy ranges from 0.14% to 2.0%. It is usually an acute event; tendon rupture often occurs within the first two weeks of treatment; however, it has been observed up to six months following discontinuation of the antibiotic [1-4].

The most common (nearly 90%) site of fluoroquinolone-associated tendinopathy is the Achilles tendon secondary to the increased load to this area during physical activity and weight-bearing; rupture complicates about 40% of drug-induced tendonitis and occurs in the body of the tendon, the site of the scantiest blood supply. Involvement is bilateral in almost 45% of cases. Tendinitis usually resolved within two months after fluoroquinolone treatment has been discontinued [1-4].

There are several risk factors for fluoroquinolone-induced tendinopathy: age older than 60 years, pre-existing tendinopathy, strenuous physical activity, concomitant glucocorticoid therapy, renal failure, renal transplantation, and hemodialysis. The pathogenesis of tendinopathy in patients who have received fluoroquinolones remains to be determined. Current hypotheses include reactive oxygen species production and accumulation (which results in apoptosis and a cytotoxic effect on extracellular matrix components) and excessive tendon loading (which results in the increased production of prostaglandin E2, interleukins, cyclooxygenase-2, and metalloproteinases) [1-4].

Statins are prescribed to treat hypercholesterolemia; they inhibit hydroxy-3-methyl-glutaryl-coenzyme A (HMG-CoA) reductase. Tendinopathy (including tendonitis and tendon rupture) is a dose-independent class adverse effect of statins occurring more commonly in men than women; the median age of the affected individuals is 56 years, and the prevalence of drug-related tendinopathy is 2.09%. Statin-associated tendinopathy has most frequently been observed with atorvastatin; however, it has also occurred in patients who have received simvastatin, pravastatin, fluvastatin, rosuvastatin, and lovastatin [1-3,5].

The median onset time of tendon injury is about eight months after starting statin treatment; indeed, 47% of the affected patients noted the problem within four months after initiating therapy. Spontaneous and substantial symptom improvement occurred after a median of 23 days after discontinuing the statin. However, tendinopathy symptoms recurred in all of the individuals who restarted the statin [1-3,5].

The Achilles tendon was affected in 52% of patients; less commonly, the following tendons, in decreasing frequency, were affected: quadriceps, epicondylar, biceps, forearm, and gluteus. The tendinopathy was either unilaterally (58.7%) or bilaterally (41.3%) and associated with sports in 15.6% of patients. Individuals with spontaneous tendon rupture tended to be older; in addition, patients with chronic kidney disease, diabetes, gout, pre-existing tendinopathy, rheumatoid arthritis, and strenuous physical activity had a higher risk of statin-associated tendon rupture [1-3,5].

Statin-associated tendinopathy was more common in patients who were concurrently receiving the following medications: cytochrome P450 3A4 (CYP3A4) inhibitors such as cyclosporin or gemfibrozil, fibric acid derivatives such as ezetimibe or fenofibrate, or other drugs associated with tendinopathy such as fluoroquinolones or corticosteroids. The pathogenesis of tendinopathy in patients receiving statins remains to be definitively established. Postulated mechanisms include the alteration of matrix metalloproteinases, decreased tenocyte migration, weakening of cell membranes (and tendon structural components) secondary to the inhibition of cholesterol synthesis, decrease in isoprenoids (resulting in musculotendinous unit weakness), and activation of apoptosis (thereby weakening the tendon matrix) [1-3,5].

Long-term (defined as greater than three months) systemic glucocorticoids, either inhaled or administered orally, can result in tendon rupture; at least 300 individuals with glucocorticoid-induced tendon rupture have been published. Patients receiving glucocorticoids who are at an increased risk of tendon injury are either those with autoimmune diseases (such as rheumatoid arthritis and systemic lupus erythematosus) or those being concurrently treated with a fluoroquinolone. In addition, in oral glucocorticoid users, the initiation of bisphosphonate therapy has been demonstrated to transiently increase the risk of tendon rupture [1-2,6-7].

The larger, lower limb tendons, such as the Achilles, patellar, and quadricipital tendons, are the primary sites of glucocorticoid-associated tendinopathy. The tendon rupture may occur from four months to several years following the initiation of drug therapy; however, in patients with chronic respiratory diseases being treated with inhaled glucocorticoid therapy, four years was the mean time from the initiation of treatment to Achilles tendon rupture. Albeit less common, spontaneous tendon rupture in the palliative care setting has been observed within two weeks after starting oral corticosteroid therapy [1-2,6].

Several hypotheses have been proposed for the mechanism of pathogenesis of glucocorticoid-associated tendon rupture. Antimitotic effects and collagenase activation by the corticosteroid exposure may deteriorate tendon collagen. Similarly, collagen synthesis may be suppressed by corticosteroid-associated suppression of tenocyte cellular activity. Finally, there may be inadequate repair of repetitive tendon microtrauma secondary to glucocorticoid inhibition of tissue repair mechanisms [2,6].

Aromatase inhibitors, antihormonal therapy used for adjuvant therapy of estrogen receptor-positive breast cancer in postmenopausal women, and first-line treatment in patients with metastatic hormone receptor-positive breast cancer, either competitively (non-steroidal aromatase inhibitors such as aminoglutethimide, anastrozole, and letrozole) or irreversibly (steroidal aromatase inhibitors such as exemestane) block the conversion of androgen to estrogen by the P450 cytochrome enzyme aromatase. In addition to musculoskeletal adverse effects, aromatase inhibitors can be associated with tenosynovitis of the fingers, hands, and wrists in up to 50% of the treated patients. Rarely, aromatase-induced severe tendinopathy and rupture of larger tendons (such as the subscapularis, infraspinatus, and/or supraspinatus tendon) have occurred [1-3,8].

Risk factors associated with aromatase inhibitor tenosynovitis include prior chemotherapy (particularly with taxanes); additional, yet controversial, possible risk factors also include prior menopausal hormone replacement therapy and obesity. The mean time of eight weeks was noted between starting the aromatase inhibitor therapy and the onset of symptoms; indeed, tenosynovitis typically occurred within three months of initiating treatment. At least in some of the affected patients, the aromatase inhibitor changes were reversible one to two years after discontinuation of the drug [2-3].

The pathogenesis of aromatase inhibitor-associated tendinopathy remains to be determined. However, estrogen receptors have been demonstrated in the intermediate layer of the annular ligaments that comprise the flexor pulley tendons and the retinaculum (bands around the tendons) of the fingers. Alteration of these tendons, based on the action of the aromatase inhibitors blocking these receptors, may result in the development of tenosynovitis [2].

In women with severe tendinopathy or rupture of the spinatus or subscapularis tendons, the onset of symptoms appeared between 11 to 18 months (median 12 months) after initiating the antihormonal therapy. The initial management included discontinuation of the aromatase inhibitors. The two patients with ruptured tendons required surgical treatment; however, the woman with severe tendinopathy and no rupture had complete resolution of her symptoms within one month after replacing her drug and conservative treatment with corticosteroids [8].

Drug-induced toxic tendinopathy has also been described, albeit less frequently, in patients who have received other systemic drugs. These include anabolic steroids, isotretinoin, antiretroviral (protease inhibitors, such as indinavir), and renin-angiotensin system-acting agents [9-12]. Also, there are single reports of tendonitis in individuals who have received amlodipine, rituximab, or sitagliptin [13-15].

There is a markedly increased risk of tendon ruptures in individuals who abuse anabolic steroids: competitive athletes, weightlifters, and recreational bodybuilders. Tendons subjected to heavy loads (such as the Achilles, bicipital, tricipital, and quadricipital tendons) are more likely to be affected; one study found that the risk of rupture was greatest for upper body tendons. The function is usually restored following surgical repair of the ruptured tendon and subsequent rehabilitation [1-2,9].

There are two hypotheses for the pathogenesis of anabolic steroid-associated tendon rupture. The first is that the drug, with or without intense muscular exercise, damages the tendon structure by altering the biomechanical properties of the tendon; hence, even in the absence of excessive stress, the tendon is more susceptible to rupture. The second is that the tendons become too weak for their corresponding muscles; the anabolic steroids cause massive hypertrophy of the muscles in the absence of concurrent strengthening of the associated tendon. Therefore, even though the drug does not have a direct detrimental effect on the tendon, it results in an increased risk of tendon rupture following acute stress [1-2,9].

Isotretinoin (13-cis-retinoic acid) is a synthetic, vitamin A-derived retinoid used in the treatment of nodulocystic acne. There are several case reports of tendinopathy (of the Achilles tendon within two to six weeks after starting the drug) and enthesopathy (characterized by premature osteophyte formation particularly along the vertebral column entheses of the cervical and thoracic spine). Isotretinoin-induced Achilles tendinopathy is considered to be reversible; interruption or termination of the medication results in regression of the Achilles tendonitis clinical symptoms [1-2,10].

Adhesive capsulitis of the shoulder has been described in patients receiving highly active antiretroviral therapy (HAART), particularly in individuals treated with protease inhibitors such as indinavir. However, tendinopathy has been observed in both HIV-negative and HIV-positive patients. A severely immunosuppressed patient developed bilateral ankle tenosynovitis six weeks after starting HAART [1-2,11].

Achilles tendinopathy was described in two men after beginning treatment with the combination protease inhibitor Kaletra (lopinavir and ritonavir). The first man developed bilateral Achilles tendonitis on the fourth day that he was receiving Truvada (tenofovir, disoproxil, and emtricitabine) and Kaletra; all of his symptoms resolved within three days after switching the Kaletra to raltegravir. The second man developed thickening and pain around both of his Achilles tendons nine months after beginning Truvada and Kaletra; within two days after changing his Kaletra to darunavir and ritonavir, his Achilles pain resolved [11].

Renin-angiotensin system-acting agents are used to treat hypertension. A statistically significant association of Achilles tendon rupture in patients treated with renin-angiotensin system-acting agents, and especially renin-angiotensin II receptor antagonists which regulate aldosterone content, was observed in a retrospective study of the National Hospital Discharge Register of all patients in Finland. Ninety-five of the 1118 Achilles tendon rupture patients from 1998 to 1999 had received renin-angiotensin system-acting agents; a potential mechanism for this adverse effect remains to be determined [12].

The Finnish study also found Achilles tendon rupture to be associated with seven other main drug groups: sex hormones, systemic corticosteroids, systemic antibiotics, anti-inflammatory agents, topical pain relievers, analgesics, and drugs for obstructive airway disease. However, patients with nonoperative treated Achilles tendon ruptures were not included in the study. In addition, the investigators commented that some of the observed associations were likely to be explained by the symptomatic management of the patient’s painful tendon prior to its rupture [12]. 

Amlodipine is a calcium channel blocker used in the treatment of hypertension. Bilateral Achilles tendonitis with tender ankle swelling and pain on walking secondary to amlodipine developed in a 50-year-old hypertensive man within two weeks after he began taking 10 mg daily; the dosage had previously been slowly increased to 10 mg a day over a period of six months. Within 10 days of stopping the medication, the tendon pain and ankle swelling resolved. A repeat challenge with 10 mg a day of amlodipine resulted in recurrence of his symptoms within three weeks; ultrasound and Doppler studies confirmed Achilles tendon thickening and acute tendonitis, respectively. Again, all of his symptoms resolved after discontinuing the amlodipine [13].

Rituximab, a mouse/human chimeric anti-CD20 IgG1 monoclonal antibody used to treat autoimmune diseases and cancer, was associated with the new onset of tendonitis in three women with connective tissue disease (systemic lupus erythematosus, rheumatoid arthritis, and antisynthetase syndrome) without preexisting tendonitis. Each woman received two doses of rituximab 1000 milligrams two weeks apart; within one week after the second dose, they developed tendonitis of their Achilles tendon (one patient), their wrist tendon (one patient) or both (one patient). Symptoms improved between three to four months after the second rituximab treatment [14].

Sitagliptin is an oral dipeptidyl peptidase-4 (DPP-4) inhibitor; it is used in the management of diabetes. Bilateral Achilles tendonitis, confirmed by magnetic resonance imaging studies, developed in a 56-year-old woman four months after restarting sitagliptin. She was maintained on the drug for an additional nine months, with persistence of her symptoms, until the temporal association of the tendonitis and restarting the drug was realized. Her Achilles pain completely resolved (on the left) and improved by 50% (on the right) within four weeks after stopping the sitagliptin [15].

Tendonitis and tendon contractures may be treated by an injection of a therapeutic agent into the surrounding tissues. However, tendon rupture has also occurred as adverse sequelae following treatment. The drugs associated with the rupture of the tendons have included collagenase *Clostridium histolyticum*, corticosteroids, and polidocanol [16-18].

Collagenase *C. histolyticum* injection, under local anesthesia, had been introduced as a potential treatment of Dupuytren’s contracture, a disorder that results in contractures of the proximal interphalangeal and metacarpophalangeal joints. The incidence of tendon rupture following collagenase *C. histolyticum* injection was 0.5%; this adverse event was usually associated with multiple injections into the affected finger. However, more recently, acute tendon rupture of both the flexor digitorum superficialis and flexor digitorum profundus of the left little finger was observed within 24 hours after a single injection of collagenase *C. histolyticum *into a palmar Dupuytren’s contracture cord of a 47-year-old right-handed man [16].

Corticosteroid injections are one of the modalities used to provide relief of tendonitis-associated pain and inflammation. However, tendon degeneration or rupture may occur following the injection. Animal studies have demonstrated that there was not only temporary induction of matrix metalloprotease-3 expression and cell apoptosis but also significantly decreased biomechanical strength one week after corticosteroid solution was percutaneously injected into the Achilles tendon-calcaneus junction; these tendon changes were not observed when the evaluation was performed three weeks following the injection [2-3,17].

Polidocanol is a sclerosing agent that is typically been used in the treatment of varicose veins. However, it has also been used in the treatment of chronic tendinopathy. The agent is injected under ultrasound guidance into the area of neovascularization around the affected tendon [18].

A 78-year-old woman presented with three months of right Achilles tendon pain; a magnetic resonance imaging documented not only chronic Achilles tendinopathy but also cystic degeneration and possible partial tearing of the middle third of the tendon. An ultrasound and color Doppler showed florid vascularity in the middle third of the tendon with multiple feeding vessels. After conservative management of her tendinopathy failed, ultrasound-guided polidocanol injection (2 mL of 5 mg per mL) to the site of maximum vascularity—the ventral aspect of her Achilles tendon between the peritenon and tendon—was performed; there was an immediate reduction in the vascularity. A similar injection was repeated one month later. Ultrasound evaluation one month after the second injection showed that her Achilles tendon had ruptured; there was a full-thickness tear of the middle third of tendon [18].

Antibiotics other than fluoroquinolones have been associated with drug-induced tendinopathy. Tendonitis or rupture of the Achilles tendon has occurred in adults receiving azithromycin or sulfonamides, respectively [12,19]. Similar to the patient described in this report, tendonitis and tendon rupture have also been observed in other individuals who have received cephalosporins [12,19-20].

The Drug Safety Research Unit in Southampton, United Kingdom evaluated the safety in everyday clinical usage of azithromycin using prescription-event monitoring during the early post-marketing period. The final cohort size of the patients treated with azithromycin was 11,275. There were no reports of tendon disorders in children treated with azithromycin; however, tendonitis and tenosynovitis were each reported in one adult within two months of the onset of azithromycin therapy [19].

A retrospective study of the National Hospital Discharge Register of all patients in Finland with Achilles tendon rupture who were surgically repaired between 1988-1999 showed a statistically significant association between the ruptured Achilles tendons and prior treatment with systemic antibiotics. The fluoroquinolones had the highest association. However, sulfonamides and cephalosporins were also associated with rupture of the Achilles tendon [12].

The Drug Safety Research Unit in Southampton, United Kingdom also evaluated 11,250 patients who received cefixime; tendonitis was observed in two adults and tenosynovitis was noted in one adult within two months of onset of cefixime therapy [19]. Japanese researchers investigated the occurrence of tendon disorders in patients who received either fluoroquinolone or cephalosporin antibiotics from the hospital formulary at the Hamamatsu University Hospital from April 1996 to December 2009. Tendon disorders were noted in three of the 17,902 patients who received cefdinir and two of the 24,864 patients who received cefcapene [20].

## Conclusions

Drug-induced tendinopathy, including tendinitis and tendon rupture, is most frequently associated with aromatase inhibitors, fluoroquinolones, glucocorticoids, and statins. In addition, other medications can also cause tendinopathy: amlodipine, anabolic steroids, antiretrovirals, isotretinoin, renin-angiotensin II receptor antagonists, rituximab, and sitagliptin. Tendon rupture has also occurred following injections of collagenase *C. histolyticum*, corticosteroids, and polidocanol. A nonagenarian developed tendinitis during treatment with cephalexin. Albeit less common, other cephalosporins, azithromycin, and sulfonamides have also been associated with toxic tendinopathy.
